# Black Soldier Fly (*Hermetia illucens*) Meal as Direct Replacement of Complex Fish Feed for Rainbow Trout (*Oncorhynchus mykiss*) and African Catfish (*Clarias gariepinus)*

**DOI:** 10.3390/life13101978

**Published:** 2023-09-28

**Authors:** Tamás Bartucz, Endre Csókás, Borbála Nagy, Márk Péter Gyurcsák, Zoltán Bokor, Gergely Bernáth, József Molnár, Béla Urbányi, Balázs Csorbai

**Affiliations:** Institute of Aquaculture and Environmental Safety, Hungarian University of Agriculture and Life Sciences (MATE), 2100 Godollo, Hungary; bartuczt98@gmail.com (T.B.); endrecsokas90@gmail.com (E.C.); nagy.borbala.3@phd.uni-mate (B.N.); gymarci97@gmail.com (M.P.G.); bokor.zoltan@uni-mate.hu (Z.B.); bernath.gergely@uni-mate.hu (G.B.); molnar.jozsef@uni-mate.hu (J.M.); urbanyi.bela@uni-mate.hu (B.U.)

**Keywords:** insect meal, replacement, rainbow trout, African catfish, direct feeding

## Abstract

In the experiments, defatted black soldier fly meal reared on vegetable byproducts was used in the fry rearing of two economically important fish species, African catfish and rainbow trout. Both fish species were reared in a recirculation system and 0–33–66–100% of the complex fry feed was replaced by a defatted prepupae meal of black soldier flies during a 28-day feeding experiment. African catfish was reared at 25 ± 1 °C while rainbow trout was reared at 12 ± 1 °C. The results showed that the growth of African catfish was not significantly reduced when 66% of the feed was replaced by soldier fly meal (mean weight in the control fish group at the end of the experiment was 0.4632 ± 0.2469 g, while the 66% group resulted mean weights of 0.4150 ± 0.1886 g) and the survival did not show any statistically different results (mean survival in control group was 57.48 ± 13.76% while it was 56.6 ± 7.763% in the 66% group). In the case of rainbow trout, replacing the feed entirely with insect meal did not cause a decrease in weight gain (final mean weight in the control group was measured at 1.9640 ± 0.4154 g, while in the group consuming only insect meal, it was 1.9410 ± 0.4248 g) or in survival (in the control group 98.5%, while in the group consuming only insect meal 99.5%). All these preliminary results indicate that black soldier fly meal can be used directly as a nursery feed in fish farming as a partial or total replacement of complete feeds. The results showed that black soldier fly meal could replace 66% of the complex brood feed of African catfish and up to 100% of rainbow trout feed without deterioration of production results. Our experiments have therefore opened the way for further experiments on insect meal in larval rearing.

## 1. Introduction

Today, aquaculture is the fastest growing food production sector. As the population grows, the demand for fish continues to increase, but aquaculture, rather than wild catches, is the main source of growth. By today, nearly 50% of fish consumed by humans is farmed [1], but this figure could rise to 60–70% by 2030 [2]. As the aquaculture sector grows, so will the demand for complete feeds. Nowadays, fishmeal is a product commonly used in animal feed. It is used because of its high protein content (around 65–70%) and excellent protein composition, but it is also rich in essential amino acids and polyunsaturated fatty acids. It is usually produced from wild, marine catches [3], and according to FAO statistics only 27% of fishmeal comes from by-products [1]. One ton of fishmeal requires about 4–5 tonnes of live fish [3,4]. Statistics from recent years show that more than 80% of the fishmeal is used in aquaculture [1]. It is clear that fishmeal production from natural aquaculture is not sustainable. Research shows that marine and ocean life has declined dramatically in recent decades, partly due to overfishing [5]. Insect protein is an excellent alternative source of protein that could replace fishmeal in the future. Although it has been experimented with in poultry feed since 1969 [5,6], it only received more attention in aquaculture production in the 2000s [7].

Nowadays, insect meal production is an intensively growing sector, mainly in China, Europe, North America, Australia, and South Asia. Currently, 16 recognised insect species can be used by producers in aquaculture [8]. Among these, the most commonly used insect species are the silkworm (*Bombyx mori* L.), the black soldier fly (*Hermetia illucens* L.), the house fly (*Musca domestica* L.), the mealworm (*Tenebrio molitor* L.), the lesser mealworm (*Alphitobius diaperinus* Panzer, 1797), the banded cricket (*Gryllodes sigillatus* (Walker, 1869)), the house cricket (*Acheta domesticus* L.) and the Jamaican field cricket (*Gryllus assimili* Fabricius, 1775)). These eight species are the most researched alternatives to fishmeal today. The nutritional value of these species is well known and their use in feed production is authorised in the European Union. The protein, amino acid, crude fat, minerals, and fatty acid composition of all species is known [8]. For reasons of space, it is not possible to present the considerable amount of information available on this subject in detail, so the following is limited to the insect species used in our experiments (the black soldier fly, hereafter referred to as BSF) and the fish species (African catfish *Clariass gariepinus* Burchell, 1822 and rainbow trout *Oncorhynchus mykiss* Walbaum, 1792).

The BSF is one of the most important and most studied insect species in recent years used for protein production [9]. It is native to tropical and subtropical areas of the Americas. Through international trade, the BSF has now reached almost every continent [9]. It can be reared well on various organic wastes and by-products [10,11,12]. The larvae reach their final size in 3–4 weeks [12]. Its use as feed has been studied in swine [13], ruminant [14], and poultry [15] production, as well as in fish production [7,16]. In feeding trials, it has been shown to be very successful in several fish species, mainly in the defatted form [8], and in some species (e.g., Atlantic salmon) it has been 100% fishmeal replacer [17,18]. Regarding the most important nutrients, black soldier fly meal composition is the following: the average protein content is 41.47% (21.6–65.5%), the average fat content of whole insect meal is 35.32% (29.4–51.53%), while the average fat content of the defatted meal is 6.96% (4.6–9.85%), while average ash content is 8.24% (2.7–13.2%) [19].

The African catfish is native to Africa and the Middle East. Its omnivorous diet includes crustaceans, insects, and fish. It grows very fast, reaching a body weight of 1 kg in half a year. The species prefers high water temperatures, the optimum breeding temperature is around 28 °C. It tolerates low water quality well. Adult fish tolerate high levels of total ammonium (NH_3_/NH_4_^+^), nitrite (NO_2_^−^), nitrate (NO_3_^−^), and low oxygen [20]. They are farmed under various conditions in different parts of the world. In Africa, catfish are mainly kept in small ponds and concrete pools, in Asia in cages, while in Europe precision farming (using flow-through and recirculation systems) is the most common breeding system. Feeding of the species in all regions is mainly based on a complete compound diet. The African catfish is one of the fish species produced in the largest quantities in the world and it is also of great importance in Hungarian fish farming [1,21].

Feeding experiments with this species have typically been conducted with fish weighing at least 0.5 g (minimum 1 month old). These experiments have typically yielded positive results: BSF can be incorporated into the diet of African catfish to a greater or lesser amount, but with success. Up to 25–50% of fishmeal in complete feed does not cause production disadvantage, but above this level, usually, problems occur [22,23,24,25,26,27] Table 1. Studies have been carried out on the direct use of different insects in the feeding of African catfish, but these have typically yielded results that are difficult to compare or incomplete [28,29,30], so these are not presented in detail here.

The rainbow trout is a species native to North America and East Asia, with subspecies inhabiting the North Pacific basin. There are also pure freshwater and anadromous forms of the species. Its diet is very diverse with freshwater populations typically consuming a variety of insects and crustaceans, while marine versions eat mainly fish and cephalopods [31]. The complete breeding technology for the species, from reproduction to commercial fish farming, was developed very early, in the late 19th century. Today it is farmed on all continents of the World except Antarctica. A wide variety of farming systems are used (cage, flow-through, and recirculation systems). In the world, nearly 1,000,000 Mt of rainbow trout are produced annually [1]. The species is demanding on water quality, having poor tolerance to high levels of ammonium and especially nitrite, it requires a significant amount of dissolved oxygen, moreover, high water temperature can be critical. The optimum breeding temperature is around 18 °C. Its feeding in closed systems relies almost exclusively on complete feed [31]. For rainbow trout experiments have been carried out to replace fishmeal by BSF [32,33]. They also investigated the effects of adding black soldier fly to complete feed on the fatty acid profile [34] and whether these effects can be modified by enriching the insects [35,36] Table 1. These studies have all demonstrated that insect meal can be an effective substitute for fishmeal. There have been well-documented direct feeding experiments with this species, in which whole, dried BSF prepupae were substituted for a proportion of the complex fish diet (33–66–100%) [37], and experiments where other insects (house crickets and superworm (*Zophobas morio* Fabricius 1776) [38] were fed in live, fresh forms by rainbow trout. The results are presented in the Discussion in comparison with our own results.

The above data showed that the potential for insect meal application exists for both species. However, it can also be seen that these studies have not been carried out on juvenile fish. This is interesting because, in terms of insect digestibility, we have data that the microbiome adapts to and aids the utilisation of insect meal feed [39,40,41,42] and there is evidence that the microbiome starts to develop in the early life stages [43]. In view of this, our research team assumed that if the larval fish can accept insect food in the early life stages, this could provide a basis for efficient insect use later on. Our preliminary experiments are therefore aimed at finding out whether very young fish, only a few days old, can consume insect food and how this affects their growth and survival. At this stage of life, the aim is not primarily to achieve better production results but to be able to use insect meal with at least the same results as conventional feed. This will allow fish to be reared using a stock already adapted to the insect meal in the high feed demand periods. Our experiment is a short (28 days) feeding study modelling a rearing phase after the start of feeding, which is well-defined in practical fish production. In this stage, the fry reaches a size that is sufficiently robust and has a significantly higher tolerance to technology through a very sensitive life stage after the start of feeding. This stage is the most knowledge-intensive stage of fish production, with low survival rates even with minor defects [44,45].

**Table 1:** Key facts from the literature on the feeding of the black soldier fly (* enriched prepupa meal; ** only the enriched meal gave good results, *** whole prepupa, direct feed).

Fish Species	Type of BSF Meal (FF: Full Fat, DF: Defatted)	Initial Size of the Stocking Material	Rate of BSF Meal (% of the Total Mixture)	Duration (Day)	The Highest BSF Rate without Negative Effect	Source
African catfish	FF	0.41–0.5 g	20–80%	84	40.3%	[22]
African catfish	FF	2.67 g	3.75–15%	48	7.5%	[23]
African catfish	FF	4 g	5.7–17.2%	60	17.2%	[24]
African catfish	FF	5–7 cm	8.75–35%	35	26.25%	[25]
African catfish	DF	5.4–6.3 g	10–30%	90%	10%	[26]
African catfish	DF	200 g	10–20%	48	20%	[27]
Rainbow trout	DF	100 g	3–15%	131	15%	[32]
Rainbow trout	-	66 g	28.1%	48	28.1%	[33]
Rainbow trout	FF *	298 g	30%	75	-	[34]
Rainbow trout	FF *	145 g	16.2–32.4%	56	32.4% **	[35]
Rainbow trout	FF *	15.3 g	1.8–18%	36	18%	[36]
Rainbow trout	FF ***	5.18 g	33–100%	90	-	[37]
Rainbow trout	FF	22.6 g	14.9–29.8%	54	25%	[38]
Rainbow trout	FF	53.39	20%	71	20%	[39]

## 2. Materials and Methods

For both species, the experiments were carried out in the recirculation fish-rearing unit of the Department of Aquaculture of the Hungarian University of Agricultural and Life Sciences. The system consisted of 30 fish tanks of 10 litres, sponge filter (TM 35 filter sponge, density:22 kg/m^3^, cell size 1600–2200 μm), UV light (3 × 15 watts), and moving bed biological filter (with a volume of 0.1 m^3^, a total biomedia surface of 800 m^2^/m^3^ and a protected surface of 600 m^2^/m^3^). The constant temperature was ensured by tailor-made PLC, which automatically switched the heating (Aqua Medic Titanium Heater 500 W) and chiller equipment (Aqua Medic Titan Chiller 500).

The African catfish was bred locally, using the institute’s own broodstock reared in a recirculation system. The fish were kept at a constant temperature of 25 ± 1 °C. Two females and two males were injected with Ovopel AUV (Interfish Ltd., Budapest, Hungary) at a dose of 1 ball/body weight. The broodstock was ovulated 12 h after treatment. The eggs and semen were stripped in a jar and swollen in Woynárovich solution (3 g urea and 4 g salt dissolved in 1 L water) for 1 h, then treated twice with 0.5 g/L tannic acid solution for 10 s. The eggs were incubated in a Zug jar at 25 ± 1 °C, and on day 3 after hatching the larvae began to feed. The larvae were fed with decapsulated *Artemia salina* cyst (Ocean Nutrition Europe) until the 6th day after the start of feeding. On day 7, the larvae were stocked in 10 l tanks at a density of 50 fish/litre (500–500 individuals per tank). Water temperature during the experiment was maintained at 25 ± 1 °C and dissolved oxygen was provided at a concentration of 6 ± 1 mg/L. The level of total ammonium (NH_3_/NH_4_^+^) was kept at 0.3 ± 0.05 mg, nitrite (NO_2_^−^) concentration was limited to 0.09 ± 0.04 mg/L, while nitrate (NO_3_^−^) level was controlled at 13.4 ± 2.4 mg/L. PH was managed at the level of 7.9 ± 0.1.

The feeding experiment lasted 28 days after a three-day feed exchange period. During the experiment, the fish were fed ad libitum (according to their appetite), 4 times a day, but each tank received the same amount of feed. The 20 tanks included in the experiment were randomly divided into 4 groups using 4 different feeding regimes in 5 replicates. The control diet consisted of a fry-rearing feed from Aller Aqua Polska Sp. z.o.o Infa (Golub-Dobrzyń, Poland), 400-micron size (64% protein, 10% fat, hereafter referred to as “control”). For the second group, 33% of the diet was replaced by defatted BSF prepupa meal (hereafter referred to as “33%”), while for the third group, this proportion was 66% (group name “66%”). For the fourth group, only pure insect meal was fed (group name “100%”). The number of replications was five for each group. The insect meal contained 41.5% protein and 8.5% fat, supplied by Agroloop Hungary Ltd. (Törökkoppány, Hungary). in the form of defatted flakes, which were powdered by hand mortar and sorted into a fraction equal to the size of the fishmeal-based fry-rearing feed. Flies were reared on suitably prepared vegetable waste. Pools were cleaned once daily throughout the experiment. During the experiment, measurements were taken at stocking and the end of the experiment on day 28. At the stocking and the end of the experiment 20–20 individuals per pool were weighed. The total length of the fish was measured from photographs using ImageJ 1.54 software (a programme developed at the National Institutes of Health and the Laboratory for Optical and Computational Instrumentation, University of Wisconsin). Body mass was measured on a Mettler Toledo AB204-S analytical balance with milligram accuracy. After weighing the wet weights, the fish were placed in a drying cabinet according to the OECD standard (OECD 210, Fish, Early-life Stage test) [46] and dried at 60 °C for 24 h then weighed again. During the experiment, changes in oxygen level, water temperature, and number of dead individuals were recorded. At the end of the experiment, the Specific Growth Rate (SGR) was calculated using the initial and final wet body weights according to the following formula:

SGR (%) = ln(W_f_) − ln(W_i_)/Δt × 100

where W_f_ is the final wet body mass, W_i_ is the initial body mass, and Δt is time in days.

In the case of rainbow trout, fish were reared at the Hoitsy and Rieger Ltd. (Lillafüred, Hungary). The propagation was carried out according to production practice. The broodstock was sorted weekly without hormone induction, and the eggs from ovulated individuals were dropped. The eggs and milt were dry mixed, water was added, and then transferred into California trays without further treatment. Incubation was carried out at 12 °C. The majority of the hatching and the non-feeding larval phase took place on the farm and was subsequently transported to the Institute. At the beginning of the feeding period, 40 fish were placed in the aforementioned pools. Again, 4 groups were formed (control, 33%, 66% 100%), with 5 replicates from each group. The control diet in this case was Aller Aqua Polska Sp. z.o.o Futura EX Gr 0.5–1 mm (60% protein, 10% fat). On veterinary advice, all groups received vitamin C supplementation (1 g/kg, BENEFITT L-ascorbic acid). Water temperature was measured at 12.3 ± 0.3 °C and oxygen content was kept at 9 ± 0.5 mg/L. The level of total ammonium (NH_3_/NH_4_^+^) was limited to 0.1 ± 0.05 mg, nitrite (NO_2_^−^) content was kept at <0.02 mg/L while nitrate (NO_3_^−^) concentration was controlled at a level of 7.25 ± 1 mg/L. The PH was managed at the level of 8.17 ± 0.1. The feeding of the fish and the measurement procedure were the same as those used in the African catfish experiments. 

For statistical analysis, ANOVA was used at a 95% significance level and with Tukey’s Multiple Comparison as a post-test, and in cases where the data did not pass on normality test (African catfish dry and wet body weight and rainbow trout dry weight), the Kruskal-Wallis test at 95% significance level as used, with Dunn’s Multiple Comparison Test as a post-test. Analyses were performed using GrapPad Prism 4.0 (Dotmatics, Boston, MA, USA).

## 3. Results

**Figure 1:**
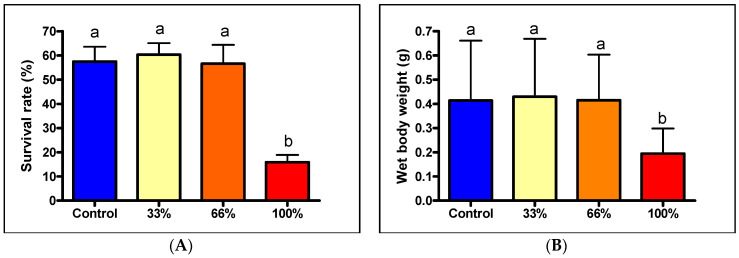
(**A**) Survival rate of African catfish fry (%) at the end of the trial (*n* = 5); (**B**) wet body weight (g) of the African catfish fry at the end of the trial (*n* = 100), different letters above the columns indicate statistically significant differences (*p* < 0.05).

In both species, the fish readily consumed BSF meal and mixed feeds, and they were not selective between different feed particles. In the case of African catfish, the mean wet body weight at stocking was measured at 0.0144 ± 0.0028 g, dry body weight 0.0018 ± 0.0006 g, and total body length 12.58 mm ± 0.7 mm. At the end of the experiment, the fish showed very similar results for the different parameters. The first parameter tested was survival. The group receiving 33% insect meal showed the best rate (60.36 ± 10.58%), but similar results were obtained for the control (57.48 ± 13.76%) and the 66% group (56.6 ± 7.763%). However, the retention of the group receiving 100% insect meal was statistically weaker (*p* < 0.001) (15.88 ± 6.798 mm). A similar trend was observed for wet weight. Fish in the control group had the highest body weight (0.4632 ± 0.2469 g); weights in the 33% group (0.429788 ± 0.239 g) and the 66% group (0.4150 ± 0.1886 g) were slightly lower, but these differences were not statistically significant, while fish in the 100% group (0.2075 ± 0.1041 g) were significantly smaller than those in the other three groups, as the post-tests showed. For dry weights, fish in the control group weighed 0.0712 ± 0.0364 g, the 33% group was 0.0711 ± 0.0424 g, the 66% group weighed 0.0645 ± 0.0317 g, while the 100% group only achieved 0.0275 ± 0.03144 g. When the total body lengths were measured, the highest value was obtained by the 33% group (36.46 ± 6.5 mm), the second was the control group (36.72 ± 5.8 mm), the third was the 66% group (35.03 ± 5.4 mm) and in this case, also the group receiving pure BSF flour was the smallest (23.82 ± 5.5 mm), which was statistically proven (Figure 1).

This also resulted in a significant divergence in growth in both body mass and length. The average increase in wet body weight for the control (0.4488 g), 33% (0.4154 g) and 66% (0.4006 g) groups was more than twice that of the group fed by pure insect meal (0.1931 g), and the same was true for dry body weight, too (C:0.0693 g; 33%:0.0963 g; 66%:0.0627 g, 100%:0.0257 g). For SGR, no such significant difference between the different groups was measured, but it can be noted that the highest value was obtained by the control (12.387%), while the worst value (9.520%) was obtained by the group receiving 100% insect meal. 

Further details are given in Table 2.

**Figure 2:**
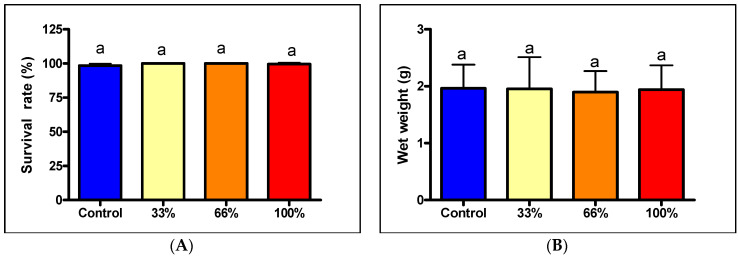
(**A**) Survival rate of rainbow trout fry (%) at the end of the trial (*n* = 5); (**B**) wet body weight (g) of the rainbow trout fry at the end of the trial (*n* = 100), different letters above the columns indicate statistically significant differences (*p* < 0.05).

The results for rainbow trout show a slightly different picture. The much larger (initial wet body weight: 0.3974 ± 0.1331 g, initial dry body weight 0.0711 ± 0.0267 g, initial total body length 34.1 ± 3.4 mm) and stronger trout fry grew slightly slower in relation to body weight, but naturally, the survival was higher (98.51–100%) than those of the much smaller larval size of African catfish (Figure 2).

The groups with different diets showed very similar survival for trout (98.51 ± 1.369–100%; *p* = 0.057). It can also be stated that hardly any statistically significant differences in body sizes among the treatments were found in the trout experiment. The *p*-values for dry and wet weights were measured as 0.7239 and 0.4167, respectively. For total body length, ANOVA found that *p* = 0.0489, however, no significant difference among the treatment groups was indicated by the post-test. The longest fish were in the control group on average (58.2 ± 4.6 mm), but the other three groups had similar average lengths (33%: 57.9 ± 5.5; 66%: 58.1 ± 4.4 mm; 100%: 56.5 ± 4.6 mm).

Net gains also showed very little variation either in wet weight (control: 1.5666 g, 33%: 1.5566 g, 66%: 1.4996, 100%: 1.5436), dry weight (control: 0.3306 g, 33%: 0.3304 g, 66%: 0.3143 g, 100%: 0.3224 g), or length (control: 24.1 mm, 33%: 23.8 mm, 66%: 24.0 mm, 100%: 22.5 mm). In all cases, the control group achieved the largest increase, although the differences were minor: no parameter showed a difference of more than 5%. The detailed data are presented in Table 3. 

## 4. Discussion

The use of insects in fish feed is a widely researched topic, but in general, limited to and typically focused only on the replacement of fishmeal [8]. Even there, it is typically used to replace only part of the fishmeal, representing 5–35% of the total feed [22,23,24,25,26,27,32,33,34,35,36,37,38,39]. This is particularly interesting in light of the fact that some fish species consume significant amounts of insects throughout their life cycle, such as African catfish [47,48,49] and especially rainbow trout [42,50,51,52]. 

In the experiment presented in this article, our aim was to investigate whether insect meal can be used directly to feed fish, including fry in the early exogenous feeding stage. The results of the experiments were conclusive, both for coldwater rainbow trout and tropical African catfish fry. In all cases, the fish responded well to the new feed ingredient, apparently not avoiding insect-derived particles, and consumed all the components of the mixture quickly and indiscriminately.

In the case of African catfish, 66% of the total diet could be replaced by insect meal without any statistically significant difference in the main parameters we studied (survival, total body length, dry and wet weight) compared to commercial feeds. This finding partly confirms the results of previous studies on fish meal replacement, where researchers obtained adequate, and in some cases superior growth parameters even at lower replacement rates [20,21,22]. It should be noted, however, that these studies used black soldier fly meal up to a maximum of 17–20% of the total mixtures and that this was also used as part of complex fish feeds. However, in the feeding regimes we studied, this percentage reached 66% and the meal was consumed directly by the animals without any other processing or mixing procedure. No comparable direct feeding experiments are known for African catfish, so we are probably the first in the world (according to our knowledge) to investigate the possibility of direct feeding of insect meal to this age class.

As already explained in the introduction, the situation is somewhat different for rainbow trout. Experiments to replace fishmeal have been successful in this case as well: fishmeal has been replaced to a greater or lesser extent by soldier fly meal, and practical fish production tests have shown that black soldier fly meal can even be used in fish farming [33]. The picture is slightly different when the whole insect is used. In an experiment conducted by Turkish researchers [37], dried black soldier fly prepupae were fed one, two, or all three times a day instead of three daily rations of complex fish feed. The results showed that even a single insect replacement had a negative effect on production parameters, and that fish not only grew more slowly at higher feeding rates but also survived less well. An interesting counterpoint is an experiment conducted in the Czech Republic [38], where, although not black soldier flies, but house crickets and superworm were used in an isocaloric feeding system. However, the results showed that there was no significant difference in the rearing of the fish between feeding a complex fish feed and feeding 100% live insects. The results of our experiment are similar to those of the latter study. Among the parameters we studied, we observed a very similar phenomenon in terms of wet and dry body weight and survival: these parameters were not negatively affected even by feeding 100% BSF meal, and the rearing results met the requirements of production practice.

Overall, although further, longer-term studies are necessary, our present results suggest that direct insect feeding of insect meal in fry-rearing may be a perspective solution in some cases. In African catfish, up to 66% of the fry-rearing feed can be replaced by this new form of feed. Rainbow trout can also be fed insect meal as a stand-alone feed. This is particularly interesting because one of the main challenges of using insect meal is the cost. While no one disputes that black soldier flies reared on waste or by-products are a sustainable feed material, the most common objection is that the high cost of such feed material makes it not worthwhile to use it at present. This is why the studies so far conclude that insects, including the black soldier fly, will become widespread in fish farming if their production reaches a level where production costs and, in turn, market prices are significantly reduced or if the price of fishmeal increases dramatically. This line of thinking is somewhat contradicted by our present study. The cost of the fish feed we used was €2.77/kg (Aller Infa) and €2.76/kg (Aller Futura). If we compare this with the price of retail BSF meal in Hungary (6.13 €) [53], it is indeed not economical, but if we look at the prices in other countries with lower production costs (0.8 €) [54], the direct use of insect meal for high priced fry rearing feeds may be a rational choice even today. 

The use of insect meal, and in particular BSF meal, is an important topic of research that is of high importance in fish farming. This type of nutrient is a sustainable and, under appropriate production conditions, economical alternative to unsustainable and increasingly expensive fishmeal. To summarise our research, the use of insect meal, and in particular direct feeding, may be a promising method for the future, but further studies are needed before practical application. In particular, it would be worthwhile to increase the duration of the study, thus shedding light on the accumulative effects. In addition, the timing of the introduction of the feed could be a very interesting issue, to allow the fish to adapt, for example, to the appropriate chitin degradation processes (enzyme activity, microbiome) [55]. Finally, several authors have already pointed out that the substrate used in BSF production can have a decisive influence on the usability of BSF meal [36,56]. It would therefore be important to investigate whether the encouraging results we have obtained are mainly due to the high quality of the insect meal or to the specific characteristics of the fish species used in the experiments. It would therefore be worthwhile to conduct further experiments to determine whether BSF grown on different substrates has such an effect on fish rearing. Finally, it is important to underline that the results obtained will be truly positive if further experiments in the future can prove that the early introduction of BSF meal has a positive effect on the whole production cycle (up to and including fish production). 

## Figures and Tables

**Table 2:** Parameters measured and calculated in the African catfish fry feeding experiment (Means of five replicate tanks. Values with different superscripts in columns are significantly different (*p* < 0.05)).

		Control	33%	66%	100%	*p*-Value
Initial	Wet weight (g)	0.0144 ± 0.0028	0.0144 ± 0.0028	0.0144 ± 0.0028	0.0144 ± 0.0028	-
	Dry weight (g)	0.0018 ± 0.0006	0.0018 ± 0.0006	0.0018 ± 0.0006	0.0018 ± 0.0006	-
	Total length (mm)	12.58 ± 0.7	12.5 ± 0.7	12.5 ± 0.7	12.5 ± 0.7	-
Final	Wet weight (g)	0.4632 ± 0.2469 ^a^	0.429788 ± 0.239 ^a^	0.4150 ± 0.1886 ^a^	0.2075 ± 0.1041 ^b^	<0.001
	Dry weight (g)	0.0712 ± 0.0364 ^a^	0.0711 ± 0.0424 ^a^	0.0645 ± 0.0317 ^a^	0.0275 ± 0.03144 ^b^	<0.001
	Total length (mm)	36.46 ± 6.5 ^a^	36.72 ± 5.8 ^a^	35.03 ± 5.4 ^a^	23.82 ± 5.5 ^b^	<0.0001
	Survival (%)	57.48 ± 13.76 ^a^	60.36 ± 10.58 ^a^	56.6 ± 7.763 ^a^	15.88 ± 6.798 ^b^	<0.001
Gain	Average wet body weight gain (g)	0.4488	0.4154	0.4006	0.1931	-
	Average dry body weight gain (g)	0.0693	0.0693	0.0627	0.0257	-
	Average total length (mm)	23.8	24.1	22.4	11.2	-
	SGR	12.387	12.119	11.994	9.520	-

**Table 3:** Parameters measured and calculated in rainbow trout fry feeding experiment (Means of five replicate tanks. Values with different superscripts in columns are significantly different (*p* < 0.05)).

		Control	33%	66%	100%	*p*-Value
Initial	Wet weight (g)	0.3974 ± 0.1331	0.3974 ± 0.1331	0.3974 ± 0.1331	0.3974 ± 0.1331	-
	Dry weight (g)	0.0711 ± 0.0267	0.0711 ± 0.0267	0.0711 ± 0.0267	0.0711 ± 0.0267	-
	Total length (mm)	34.1 ± 3.4	34.1 ± 3.4	34.1 ± 3.4	34.1 ± 3.4	-
Final	Wet weight (g)	1.9640 ± 0.4154 ^a^	1.9540 ± 0.5582 ^a^	1.8970 ± 0.3691 ^a^	1.9410 ± 0.4248 ^a^	0.7239
	Dry weight (g)	0.4017 ± 0.1062 ^a^	0.4015 ± 0.1338 ^a^	0.3854 ± 0.0885 ^a^	0.3935 ± 00.1337 ^a^	0.4167 ^a^
	Total length (mm)	58.2 ± 4.6 ^a^	57.9 ± 5.5 ^a^	58.1 ± 4.4 ^a^	56.5 ± 4.6 ^a^	0.0489 ^a^
	Survival (%)	98.51 ± 1.369 ^a^	100 ^a^	100 ^a^	99.5 ± 1.118 ^a^	0.0517 ^a^
Gain	Average wet body weight gain (g)	1.5666	1.5566	1.4996	1.5436	-
	Average dry body weight gain (g)	0.3306	0.3304	0.3143	0.3224	-
	Average total length (mm)	24.1	23.8	24.0	22.5	-
	SGR	5.706	5.688	5.582	5.664	-

## Data Availability

The data presented in this study are available on request from the corresponding author.
